# Polymerase face-off: emerging concepts in transcription-replication coordination

**DOI:** 10.1038/s44319-026-00800-w

**Published:** 2026-05-15

**Authors:** Sidrit Uruci, Maxime Lalonde, Martijn S Luijsterburg, Stephan Hamperl

**Affiliations:** 1 Department of Human Genetics, Leiden University Medical Center, Leiden, The Netherlands; 2 Institute of Epigenetics and Stem Cells, Helmholtz Center Munich, Munich, Germany

**Keywords:** Chromatin, Transcription & Genomics, DNA Replication, Recombination & Repair

## Abstract

Transcription–replication conflicts (TRCs) arise when DNA replication forks encounter actively transcribing RNA polymerases, creating a major threat to genome stability. These conflicts, which can occur in different orientations, disrupt replication fork progression, impair transcriptional fidelity and reshape the chromatin landscape. In this review, we discuss emerging conceptual insights into how cells coordinate replication and transcription in space and time to minimise such encounters, and we highlight the central role of RNA polymerase II dynamics in both preventing and resolving TRCs. We further describe how TRCs engage a broad network of genome maintenance pathways that regulate R-loops, stabilize stalled forks and maintain chromatin integrity. Importantly, elevated or mismanaged TRCs create vulnerabilities that many cancers exploit, positioning conflict-resolution mechanisms as attractive therapeutic targets. Finally, we examine current challenges in detecting and analysing these transient, dynamic events and underscore the need for improved imaging and sequencing technologies to study the genome’s molecular “traffic jams”. A deeper mechanistic understanding of TRCs will be crucial for harnessing them in precision oncology and clarifying their broader roles in genome regulation.

## Abstract (web summary)

This review discusses how cells coordinate replication and transcription in space and time to minimise conflicts, focusing on the role of RNAP II and other factors, how they act to maintain chromatin integrity, and how these dynamic events can be studied and new insight exploited for therapy.

## Graphical abstract

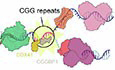

## Introduction

The diploid human genome comprises over 6 billion base pairs and approximately 40,000 protein-coding gene copies, representing two alleles of the ~22,000 unique genes (Venter et al, 2001). All DNA must be replicated once per cell cycle, whereas most genes are transcribed multiple times. Moreover, as a result of pervasive intergenic and non-coding transcription, nearly 85% of the genome exhibits detectable transcriptional activity, albeit of different levels at non-coding sequences and protein-coding genes (Hangauer et al, 2013). Replication and transcription must therefore be tightly regulated DNA metabolic processes. Extensive evidence from both prokaryotes and eukaryotes shows that encounters between DNA and RNA polymerases can cause replication fork arrest, transcription stalling, DNA breaks, recombination intermediates and mutations, ultimately threatening genome integrity (Garcia-Muse and Aguilera, 2019). Consequently, mechanisms that limit interference between replication and transcription are essential for faithful genome duplication and ensure transcriptional fidelity.

**Figure 1:**
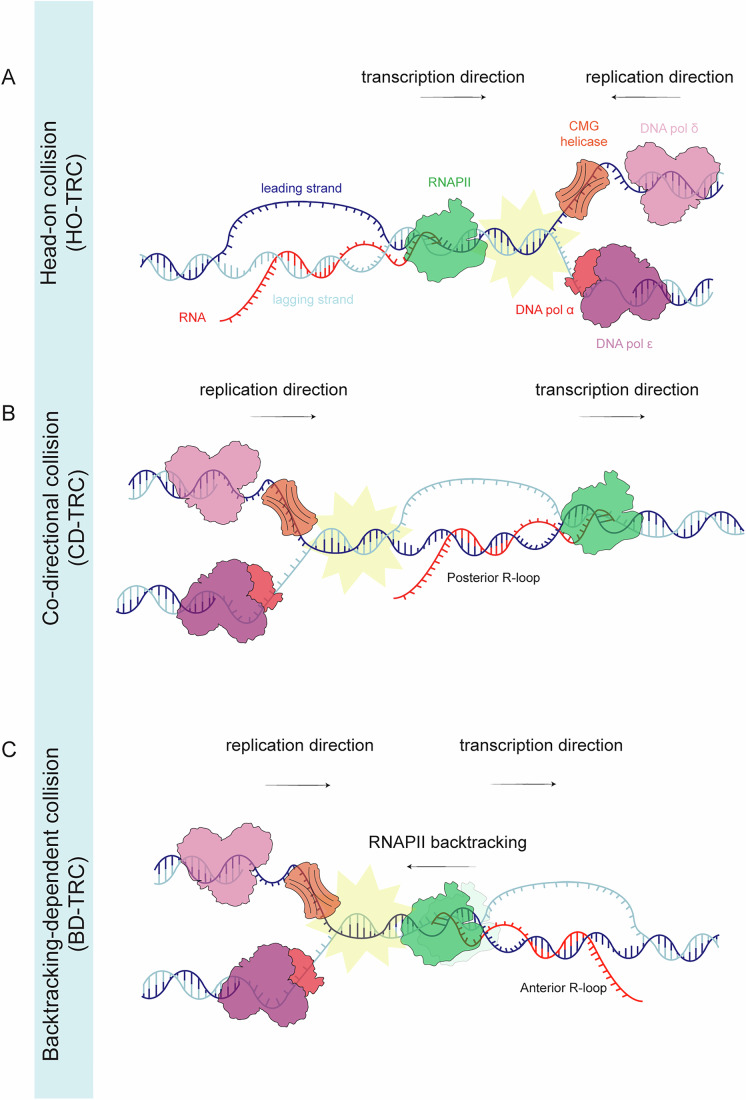
Orientation of transcription-replication conflicts (TRCs). (**A**) Head-on (HO) TRCs occur when the replication fork collides in a frontal orientation with RNAPII transcribing on the lagging-strand template. (**B**) Co-directional (CD) TRCs arise when the replication fork collides in the same directionality with RNAPII transcribing on the leading-strand template. This type of collision can also occur when the replication fork collides with an R-loop generated behind RNAPII. (**C**) Backtracking-dependent (BD) TRCs are complex collisions in which replication and transcription move along the DNA template in the same direction, but due to RNAPII backtracking, the replisome encounters RNAPII in a frontal fashion leading to a CD-like collision but with a HO-like barrier.

Because both processes proceed with 5′ → 3′ polarity, transcription-replication conflicts (TRCs) are classified based on the orientation of RNA polymerase II (RNAPII) relative to the replication fork (Hamperl et al, 2017; Hamperl and Cimprich, 2016). Head-on (HO) TRCs arise when transcription occurs on the lagging-strand template, placing the replisome opposite the transcription machinery and leading to a frontal collision (Fig. 1A). In contrast, co-directional (CD) TRCs occur when RNAPII transcribes genes on the leading-strand template, moving in the same direction as replication (Fig. 1B) (Garcia-Muse and Aguilera, 2019; Lalonde et al, 2021; Lin and Pasero, 2017; Uruci et al, 2021; Yakoub and Luijsterburg, 2023). Although both configurations are deleterious, HO collisions are more strongly associated with genome instability (Corazzi et al, 2024; Hamperl et al, 2017; Paul et al, 2013; Yakoub and Luijsterburg, 2023). Indeed, single-molecule optical tweezer experiments using reconstituted eukaryotic machineries show that RNAPII acts as a polar roadblock specifically in HO orientation, causing replisome stalling, RNAPII backtracking, R-loop accumulation and elevated topological stress (Kay et al, 2025).

To reduce HO-TRCs, bacterial genomes preferentially co-orient highly transcribed and essential genes with replication forks (Atre et al, 2024; Lang et al, 2017). This orientation minimizes situations in which the replisome directly encounters an opposing transcription complex: a configuration known to strongly destabilize genome integrity. However, this protective strategy inevitably increases the frequency of CD-TRCs, in which the replisome follows behind RNA polymerase complexes on the same DNA template. In eukaryotes, a similar logic applies. Replication origins in human cells are frequently positioned at transcription start sites (TSSs), making early encounters between the replisome and RNAPII highly likely but predominantly co-directional (Chen et al, 2019). This arrangement helps to limit the formation of deleterious HO-TRCs. Although generally considered less harmful, CD collisions can still impede replication, particularly at highly transcribed loci or when co-transcriptional obstacles arise, such as backtracked RNAPII complexes (Zatreanu et al, 2019), altered DNA supercoiling (Chedin and Benham, 2020; Hwang et al, 2025), or the formation of secondary structures like G-quadruplexes (Batra et al, 2025; Sato et al, 2025), and R-loops (Kay et al, 2025).

Importantly, the distinction between HO and CD configurations is not always straightforward. Because CD-TRCs are far more common, some conflicts initially classified as HO may instead represent cases where a co-directional replisome encounters a backtracked RNAPII, functionally mimicking an HO-like barrier, a backtracking-dependent conflict (BD-TRC) (Fig. 1C). Recent work has identified CD-specific pathways that mitigate these events (Bhowmick et al, 2023; Kloeber et al, 2025; Uruci et al, 2026), overturning earlier assumptions that CD-TRCs are benign (Lang et al, 2017) or even beneficial (Brambati et al, 2018).

This review summarizes recent advances in our understanding of TRCs, focusing on how these collisions destabilize genome integrity. We discuss the molecular mechanisms involved in conflict sensing and resolution, with particular emphasis on the emerging transcription-centered role of RNAPII regulation and dynamics and their effects on transcription and the underlying chromatin. Finally, we highlight current challenges and advances in studying TRCs, underscoring their pathological relevance in cancer.

## Molecular mechanisms of conflict sensing and resolution

### Transcription-replication coordination

**Figure 2:**
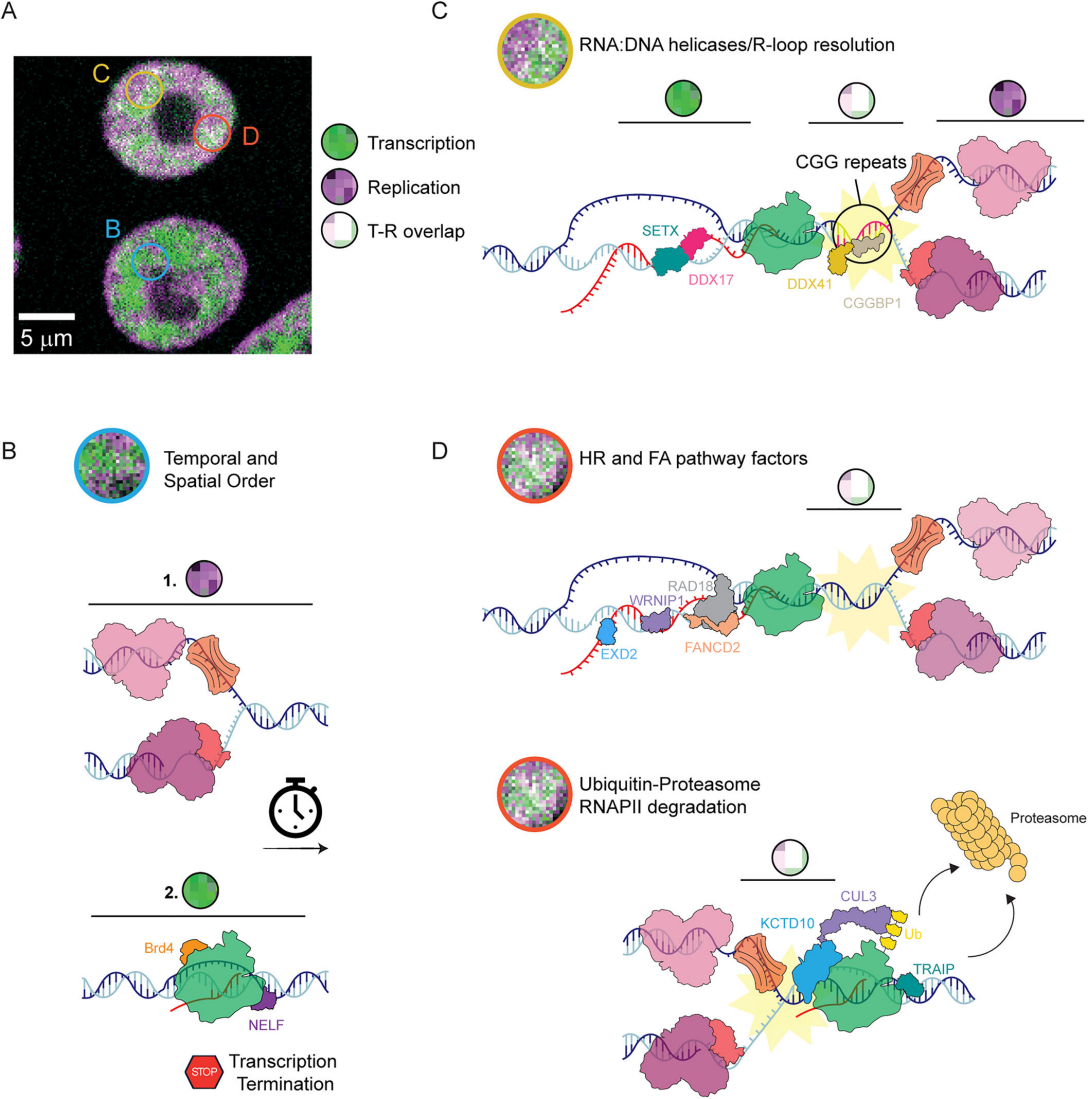
Molecular mechanisms of TRC coordination and resolution. (**A**) Representative immunofluorescence image of human pluripotent stem cells labeled for elongating RNAPII (RNAPII-pS2, in green) and nascent DNA synthesis (EdU, in magenta). The image illustrates the close spatial proximity between active transcription and replication domains during S phase, underscoring regions where transcription and replication are well coordinated (**B**) or co-occurring (**C**, **D**). (**B**) Temporal (1) and spatial (2) organization of replication and transcription machineries allow the avoidance of conflicts, by delaying or restricting transcription to prioritize replication. (**C**) RNA:DNA helicases such as DDX17 and DDX41 have a central role in R-loop resolution to resolve TRCs. Working in concert with either SETX or specialized sequence-specific binding proteins (CGGBP1), they unwind the RNA:DNA hybrid. (**D**) (top) Homologous recombination (HR) and Fanconi anemia (FA) factors are also recruited at difficult-to-replicate and R-loop-prone regions to protect replication forks from breakage and to allow fork restart, while direct biochemical resolution of R-loops is needed for the resolution of the collision (bottom). When collisions cannot be avoided or resolved, proteasome-dependent degradation of RNAPII is used to allow replisome bypass of the barrier. See review text for more information on the roles of the depicted factors.

The widespread nature of transcription and replication requires extensive spatial and temporal coordination of the two machineries to prevent TRCs and maintain genome integrity (Fig. 2A). The inherent bursting dynamics of transcription (Rodriguez et al, 2019) help mitigate the risk of TRCs by introducing intermittent periods of reduced transcriptional activity. Similarly, the preferential positioning of replication origins upstream of active genes further limits head-on collisions (Chen et al, 2019; Petryk et al, 2016; Promonet et al, 2020). Despite these spatial and temporal safeguards, such mechanisms are insufficient to fully prevent conflicts. Indeed, recent studies highlight multi-layered “traffic management” at different genomic scales, involving spatial confinement into distinct compartments, modulation of elongation kinetics of the two machineries and, where necessary, the timely stabilization and remodeling of replication forks and the displacement of RNAPII from the DNA template. At collision sites, this process is promoted by checkpoint signaling and chromatin remodeling, mediated by Mec1/ATR-dependent checkpoint activation, the INO80 chromatin remodeling complex, and the p97 segregase, which collectively facilitate RNAPII removal (Poli et al, 2016).

An effective strategy for avoiding collisions is to impose a temporal order on when replication and transcription are allowed to occur. A recent example of this strategy was described for the rapid nuclear divisions of *Drosophila* embryos, where the onset of transcription is delayed until replication has proceeded, allowing RNAPII to transcribe behind advancing replication forks (Cho et al, 2022). Mechanistically, the co-activator Brd4 bookmarks sites of active transcription on mitotic chromosomes and recruits the replication activator Cdc7, thereby prioritizing replication initiation and temporarily delaying the assembly of new Brd4 transcription hubs (Fig. 2B) (Cho and O’Farrell, 2025).

Cells also maintain spatial coordination of the two machineries. This is partially enforced by the negative transcription regulator NELF (specifically NELF-C), which plays a critical role in proper transcription termination, preventing “read-through” RNAPII transcription elongation into replication initiation zones (Fig. 2B) (Nakayama et al, 2026). Live-cell microscopy in yeast shows a reduction in transcription, kilobases ahead of the approaching replisome, suggesting local coordination at individual loci surrounding traveling forks (Tsirkas et al, 2022).

Nevertheless, a recent genome-wide approach showed that replication and transcription machineries can sometimes co-exist in close proximity, and in some instances, transcription can even facilitate replication progression by reducing histone density (Rojas et al, 2024). However, highly transcribed genes often exhibit increased replication fork stalling and DNA damage, overlapping with fragile regions and cancer-associated breakpoints (Rojas et al, 2024), indicating that TRCs are hotspots for replication stress and genome instability.

To mitigate these risks, cells exhibit remarkable plasticity. Actively transcribing RNA polymerases can push multiple MCM helicases from licensed origins over long distances, even in the context of chromatin (Scherr et al, 2022). This phenomenon, first demonstrated in vivo in yeast (Gros et al, 2015), likely contributes to the deregulated firing of intergenic origins observed in cancer cells upon oncogene-induced shortening of G1 phase (Macheret and Halazonetis, 2018). This mechanism provides a simple but powerful way to redistribute origins of replication away from highly transcribed genes, thereby limiting initiation within gene bodies. Transcription in the G1 phase may also have a preventive role prior to S phase entry. Combined with spatial and temporal coordination, this inherent mobility of replication origins ensures successful chromosome duplication despite dense molecular traffic on the DNA template.

### R-loop prevention at trinucleotide repeat structures and non-coding RNA loci

The principal hotspots for TRCs are genomic loci that are highly expressed and therefore show high occupancy of R-loops and/or RNAPII molecules. However, certain repetitive DNA elements form additional secondary DNA and RNA structures, and cells require specialized protein complexes to coordinate transcription and replication on such more challenging DNA templates.

At short CGG repeats, the CGG-binding protein 1 (CGGBP1) acts as a sequence-specific safeguard by recruiting the RNA:DNA helicases DDX41 and DHX15 to unwind R-loops, preventing RNAPII stalling and TRCs (Fig. 2C) (Ummethum et al, 2025). Similarly, at CAG repeats, the THO complex (via Thp2) and the TRAMP complex (via Trf4) work synergistically. THO prevents R-loop-induced DNA fragility, whereas TRAMP degrades unprocessed RNA to maintain RPA availability, thereby mitigating replication stress (Brown et al, 2022).

Beyond triplet repeats, ZC3H4, a component of the Restrictor complex, maintains stability at non-coding RNA (ncRNA) loci by suppressing hyper-transcription bursts. By restricting ncRNA production, ZC3H4 prevents the formation of R-loops and TRCs that can otherwise drive DNA damage and senescence (Frey et al, 2025). Collectively, these mechanisms demonstrate that preventing R-loop-mediated replication blockage requires a combination of sequence-specific binding, active helicase recruitment and global RNA surveillance.

### R-loop helicases Sen1/SETX and other DEAD-box helicases

One of the key regulators of conflicts in yeast is the conserved helicase Sen1, which removes stalled RNAPII at TRC sites (Aiello et al, 2022). In this context, Sen1 and its human homolog Senataxin (SETX) have dual functions in facilitating transcription termination (discussed in Section 3.3) and resolving RNA:DNA hybrids during S-phase to prevent TRCs. For the latter function, Sen1/SETX was shown to directly interact with active replication forks and remove RNA:DNA hybrids in the path of the replisome to prevent fork stalling and nascent DNA degradation (Fig. 2C) (Rao et al, 2024). Other DEAD-box helicases cooperate with SETX and complement this pathway. DDX17 associates with R-loops and works alongside SETX within the MUS81–LIG4–ELL cleavage and ligation pathway (Boleslavska et al, 2022). In this context, DDX17 unwinds the R-loop to allow MUS81-dependent fork cleavage and subsequent restart (Fig. 2C) (Rao et al, 2024). Interestingly, while R-loops are often genotoxic, R-loop formation at stalled forks has been suggested to contribute to the protection of nascent DNA from DNA2-mediated degradation (Song et al, 2025). However, in an alternative model, the R-loops at stalled forks facilitate DNA2-mediated DNA resection and allow proper fork restart (Xu et al, 2025). In line with this model, post-replicative R-loops have also been implicated in impairing fork restart in RNase H2-deficient contexts (Heuze et al, 2023).

The importance of these helicases is also highlighted in oncogenic contexts. For instance, SETX is essential for managing replication stress induced by the MYC oncogene. Loss of SETX upon MYC activation causes selective DNA damage at TRC-prone regions, creating a genetic vulnerability that may open new therapeutic options in cancer therapy (Sberna et al, 2025). Collectively, these helicases ensure that R-loops are either prevented or resolved to maintain replication fork progression and promote genome stability.

### Recruitment of homologous recombination and Fanconi anemia factors to TRC sites

Several studies suggest that maintaining genome integrity at TRC sites requires the coordinated recruitment of homologous recombination (HR) and Fanconi anemia (FA) pathway factors. Genomic regions replicated and transcribed in early S phase are particularly vulnerable to TRCs and rely on the key HR recombinase RAD51 to suppress replication fork breakage and protect these high-traffic loci from cancer-associated rearrangements (Bhowmick et al, 2022). Consistently, RAD52, an indispensable partner for RAD51 loading, was recently shown to interact with the transcription machinery and facilitate R-loop dissolution at TRC sites (Jalan et al, 2024). Similarly, BRCA2 acts as a critical suppressor of HO-TRCs by recruiting RNase H2 to resolve toxic R-loops (Goehring et al, 2024). When BRCA2 is deficient, under-replicated DNA persists into mitosis, triggering mitotic DNA synthesis (MiDAS) at sites that represent hotspots for rearrangements in breast tumors (Groelly et al, 2022).

When primary resolution mechanisms, such as helicase SETX or HR factors, fail, the FA pathway serves as a vital backup, particularly in rapidly proliferating cells like primordial germ cells (PGCs) (Yang et al, 2022). In this context, core FA components such as FAAP100 and FANCE are required to resolve co-transcriptional R-loops and protect replication forks. Loss of these factors leads to FA pathway inactivation, TRC accumulation and p53-mediated depletion of primordial germ cells (Xu et al, 2023b; Zhou et al, 2023). Mechanistically, the recruitment of FA factors is mediated by the E3 ubiquitin ligase RAD18, which facilitates the localization of FANCD2 to difficult-to-replicate, R-loop-prone sites (Fig. 2D) (Wells et al, 2022). In SETX-deficient cells, FANCD2 promotes the resolution of under-replicated DNA in mitosis via ERCC1-XPF and MUS81 endonucleases, ensuring successful chromosome segregation (Said et al, 2022). Collectively, these pathways prevent the conversion of TRCs into double-strand breaks (DSBs), safeguarding against chromothripsis and the complex genomic rearrangements characteristic of advanced cancers (Techer and Pasero, 2025).

### Sensing and resolution factors and pathways

Beyond these primary DNA repair pathways, several diverse sensing and resolution pathways have been discovered that ensure genome stability during TRCs. The CUL3^KCTD10^ E3 ligase complex acts as a molecular sensor that detects CD-TRCs. By bridging the replisome and transcription machinery, it recruits CUL3 to ubiquitylate and remove the TFIIS-like elongation factor TCEA2, effectively remodeling RNAPII to allow replisome bypass (Fig. 2D) (Kloeber et al, 2025). Similarly, the TRAIP E3 ubiquitin ligase is essential during S phase to prevent DNA damage at TSSs, acting as a safeguard against early S phase collisions (Fig. 2D) (Scaramuzza et al, 2023).

Ubiquitin-mediated regulation is further supported by factors such as UBE2T, which ubiquitylates the FANCD2–FANCI complex. This activation is vital for resolving R-loops in large genes and promoting mitotic DNA synthesis (Yu et al, 2023). Additionally, WRNIP1 utilizes its ubiquitin-binding zinc finger domain to localize to R-loops and transcription complexes, facilitating replication restart after transcription-induced stalling (Fig. 2D) (Valenzisi et al, 2024). For direct biochemical resolution, EXD2 serves as a specialized R-loop resolvase. Recruited by PARP1-synthesized PAR polymers and stabilized by acetylation, EXD2 preferentially degrades the RNA strand within hybrids (Fig. 2D) (Li et al, 2025). Together, these pathways illustrate a sophisticated network of ubiquitylation, enzymatic remodeling and direct RNA degradation that prevents the pathological persistence of TRC-induced R-loops and associated genomic instability.

## The role of RNA polymerase II dynamics in mitigating TRCs

### Regulating transcription initiation and proximal-pause sites

Although regulation of replication origins and co-directional transcription is essential, control of bidirectional transcription at promoters also has a significant impact on TRCs, as antisense transcription can encounter the replisome head-on. After two pre-initiation complexes (PICs) are assembled in opposite orientations around promoters, the multicomponent Integrator complex has a critical role in selecting the orientation for transcription through its INTS11-dependent cleavage function (Eaton et al, 2024; Yang et al, 2024a). This “decision” can be driven by multiple factors. One is the uneven distribution of U1 snRNA-binding sites, which are more common in coding regions than in promoter upstream transcripts (PROMPTs). The absence of U1 snRNA-binding sites at PROMPTs drives termination of antisense transcription (Eaton et al, 2024; Lykke-Andersen et al, 2021; Yang et al, 2024a). In addition, CDK9 activity, presumably through SPT5 phosphorylation, leads to INTS11 dissociation, favoring genic transcription in the sense direction (Cortazar et al, 2019; Eaton et al, 2024; Fianu et al, 2021). Interestingly, single-cell full-length nascent RNA sequencing suggests that antisense and genic, sense transcription of the same gene rarely co-occur in the same cell (Ma et al, 2025).

**Figure 3:**
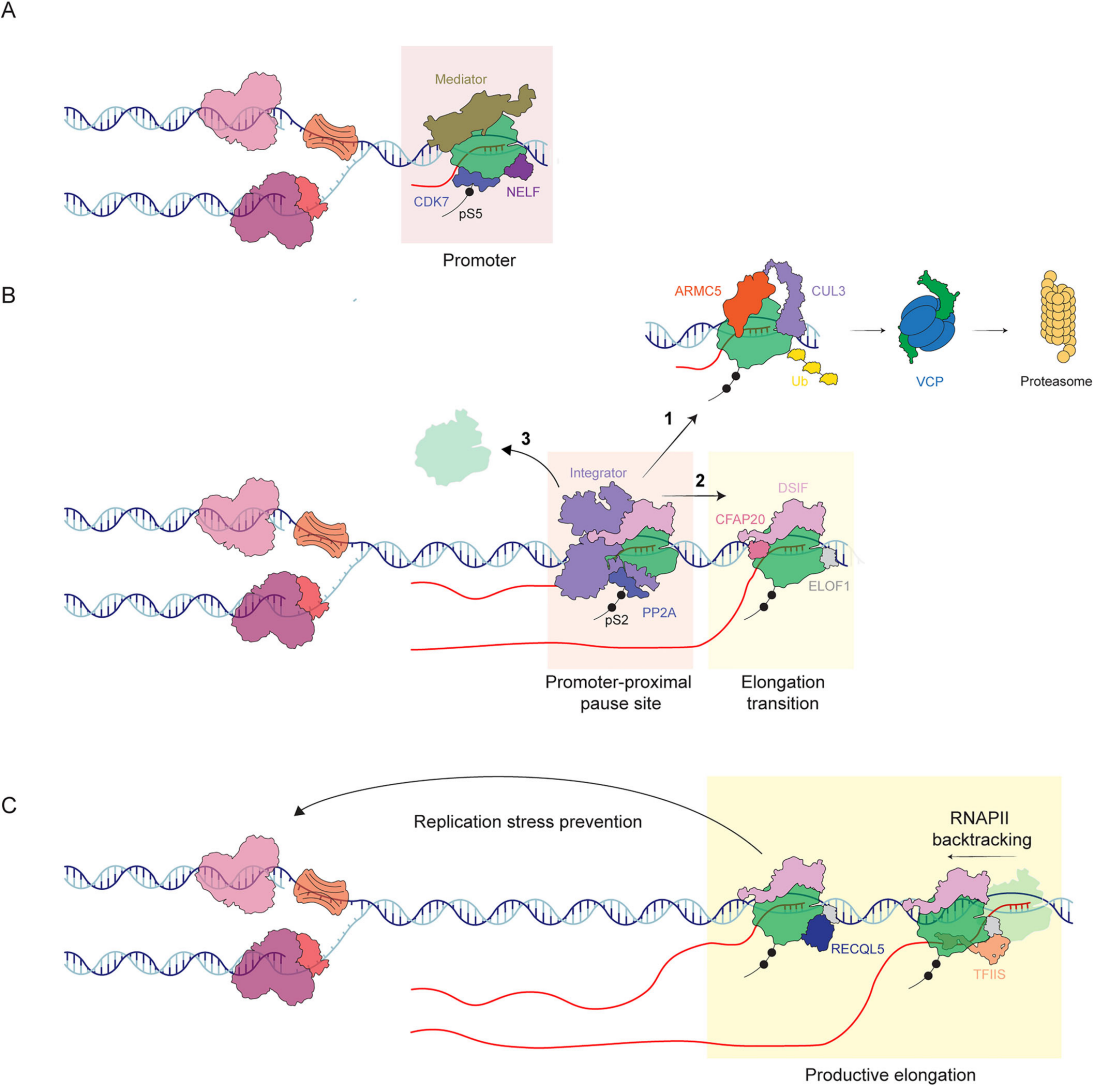
RNAPII dynamics in conflict regulation at different stages of transcription. (**A**) At promoter-proximal regions, RNAPII initiates transcription at a relatively slow rate, constituting a potential obstacle for incoming replication forks and increasing the likelihood of TRCs in these regions. (**B**) After promoter release, RNAPII pauses near promoter-proximal sites and must be processed to allow replisome passage. (1) If RNAPII cannot be restarted and pushed into productive elongation, CUL3^KCTD10^ mediates p97/VCP-dependent degradation of RNAPII. (2) In contrast to the Mediator-dependent high flux of RNAPII at promoter-proximal sites, CFAP20 rescues arrested RNAPII engaged in R-loops by promoting entry into the elongation phase. (3) If RNAPII cannot be restarted and transcription is defective, the Integrator complex removes RNAPII together with the nascent transcript to prevent collisions. (**C**) Once RNAPII enters the elongation phase, factors such as RECQL5 can act as negative elongation regulators, slowing transcription to reduce replication stress from incoming forks. Proper regulation of RNAPII backtracking is also essential for restoring productive elongation and minimizing collisions.

Following initiation of sense transcription, RNAPII frequently pauses at promoter-proximal sites as a regulatory checkpoint before productive elongation (Zhu et al, 2025). Recent studies have focused on the regulation of these sites, as their dysregulation could lead to increased TRCs (Fig. 3A). Integrator, and in particular INTS11, is recruited globally to paused RNAPII at promoter-proximal sites as a mechanism to prevent the release of paused RNAPII into faulty elongation (Lykke-Andersen et al, 2021; Stein et al, 2022), a mechanism that is antagonized by nascent RNA methylation (N6-methyladenosine (m6A)) driven by the METTL3/METTL14 complex to regulate gene expression (Xu et al, 2022).

A recent study using genome-wide approaches and rapid, selective inhibition of CDK7 suggests that this kinase is responsible for establishing pausing at promoter-proximal sites (Fig. 3B) (Velychko et al, 2024). Upon timely CDK7 inhibition, initiation factors and the Mediator co-activator complex remain associated with RNAPII, skipping the pause at the +1 nucleotide. This still allows promoter escape of RNAPII with Mediator and initiation factors attached, which compete with elongation factors and lead to an initially slow elongation rate. Upon downstream phosphorylation of RNAPII’s CTD, cells enter normal elongation, although cell proliferation is altered (Velychko et al, 2024). These results are in line with recent structural studies of RNAPII elongation complexes (Chen et al, 2021; Garg et al, 2023; Vos et al, 2018).

In addition, a novel pathway has been described through the identification of ARMC5, a CUL3 adapter. The CUL3^ARMC5^ complex, through its interaction with CDK9, mediates a non-canonical p97/VCP-dependent degradation of promoter-proximally paused RNAPII lacking the elongation factor SPT5, resulting in early transcription termination (Fig. 3B) (Aoi et al, 2025). This pathway, driven by RPB1 ubiquitylation of the phosphorylated CTD-serine 5 to induce degradation, is also triggered by defects or absence of capping enzymes and Integrator (Blears et al, 2024). CUL3^ARMC5^ appears to function in parallel with Integrator, since loss of CUL3^ARMC5^ and of the phosphatase function of Integrator (INTS6 and INTS8) leads to uncontrolled release of incompetent RNAPII into elongation, culminating in RNAPII pausing within gene bodies and generating incomplete, poor-quality transcripts (Blears et al, 2024; Cacioppo et al, 2024). It is intriguing to speculate about a possible role of CUL3^ARMC5^ in avoiding CD-TRCs, although more studies are required.

The roles of CDK7, CDK9 and CUL3^ARMC5^ have not yet been directly studied in relation to TRCs. By contrast, Integrator specifically suppresses CD-TRCs at promoter-proximal sites, thereby safeguarding genome stability. (Bhowmick et al, 2023). Proteomics experiments identified Integrator in association with both replication forks and RNAPII independently, supporting its role in resolving conflicts, presumably through its INTS11-dependent endonuclease activity. Integrator thus may prevent deleterious consequences of CD-TRCs by removing RNAPII from chromatin and resolving R-loops (Fig. 3B) (Bhowmick et al, 2023).

To further characterize regulation of TRCs at promoter-proximal sites, a recent study used a combination of CRISPR screens and genomics approaches, identifying CFAP20 as a novel factor involved in a protective pathway. CFAP20 salvages arrested RNAPII engaged with an R-loop at promoter-proximal sites, thereby avoiding CD-TRCs with incoming replisomes (Uruci et al, 2026). Through a genetic interaction with the Mediator complex, CFAP20 helps release a high flux of RNAPII that creates transcriptional stress and induces R-loop accumulation, leading to replication fork stalling *in cis* and fork speeding in trans. In CFAP20-depleted cells, the roadblock for replisomes is not the stalled RNAPII itself but RNAPII engaged with R-loops (Fig. 3B). As CFAP20 is a small, non-enzymatic protein, it is unlikely to directly coordinate CD-TRCs. Further investigation of the CFAP20 interactome is required to dissect other factors potentially involved. Interestingly, the double loss of CFAP20 and Integrator showed an additive phenotype, revealing that they represent distinct, parallel mechanisms for CD-TRC prevention (Uruci et al, 2026).

Another mechanism of TRC and R-loop regulation through Integrator is driven by the single-stranded DNA exposed during R-loop formation at promoter-proximal sites, which is recognized by SOSS, a heterotrimeric DNA damage and repair-sensing complex that contains INTS3. This leads to recruitment of the Integrator complex alongside the PP2A enzyme (Fig. 3B) (the INTAC complex) (Zheng et al, 2020), followed by condensate formation at promoter-associated R-loops that are processed by exonucleases to maintain genome stability (Xu et al, 2023a). This study adds a layer of complexity to promoter-proximal site regulation, warranting future work on the biophysical properties of these sites.

### Regulating transcription elongation and RNAPII backtracking

After efficient regulation at promoter-proximal sites, RNAPII engages in elongation with the help of several elongation factors (van der Weegen et al, 2021; Wang et al, 2022; Zhang et al, 2025a; Zhang et al, 2025b) to counteract obstacles to the elongation process (Mohamed et al, 2022; Noe Gonzalez et al, 2021). Recent structural and genetic studies have focused on the role of RECQL5 in regulating transcription elongation. RECQL5 is a DNA helicase previously identified as a regulator of transcription elongation and genome stability (Saponaro et al, 2014; Urban et al, 2016). Recent structural work highlights how RECQL5, through a direct interaction with RNAPII via a newly identified α-helix, acts as a brake on fast-elongating transcription, triggering recruitment of the transcription-coupled repair (TCR) complex (Fig. 3C) (van den Heuvel et al, 2021; van der Meer and Luijsterburg, 2025; van Sluis et al, 2025) to probe for potential DNA damage (Florez Ariza et al, 2025; Zhang et al, 2025b). This is a regulatory mechanism that restrains uncontrolled RNAPII elongation, which would otherwise disrupt co-transcriptional processes such as splicing and compromise genome stability. In line with this, RECQL5 has also been studied in a transcription-independent role in restricting RAD51-mediated excessive fork reversal at stalled forks to promote DNA synthesis (Fig. 3C) (Nagraj et al, 2025), placing RECQL5 as a key player in TRC regulation (Urban et al, 2016).

Similar to RECQL5, other factors directly bind elongating RNAPII to regulate this process. WRNIP1, through HUWE1-dependent ubiquitylation, suppresses ATM activation during an unperturbed cell cycle, thereby avoiding TRCs (Einig et al, 2023). Additionally, CDK12 is recruited to sites of MYC-induced damaged DNA via both PARP-dependent DNA damage response signaling and the SPT5 subunit of elongating RNAPII, where it suppresses transcription and thus prevents TRCs (Curti et al, 2024).

Another important regulatory event in transcription elongation is RNAPII backtracking, in which the 3′ end of the nascent RNA disengages from the active site, resulting in transcriptional pausing and arrest (Cheung and Cramer, 2011; Nudler, 2012). The anti-backtracking factor TFIIS cleaves the extruded RNA, resuming proper elongation, avoiding accumulation of anterior R-loops and maintaining spliceosome fidelity (Fig. 3C) (Zatreanu et al, 2019). A recent study using a long-range cleavage sequencing method (LORAX-seq) showed that RNAPII can backtrack by ~20 nucleotides, leading to persistent backtracking. Importantly, these events do not occur randomly in the genome but show a clear preference towards promoter regions and intron–exon junctions, and are more frequent in genes involved in the cell cycle, development and nucleosome assembly (Yang et al, 2024b). This broadens the concept of TRCs, prompting future research to consider the relationship between TRCs, the RNAPII state, and the impacts on splicing. Accordingly, a recent study employing next-generation sequencing showed how, after chromatin replication, RNAPII can resume transcription on nascent DNA and collide with the replication fork traveling ahead, impacting alternative splicing patterns (Bruno et al, 2024).

### Regulating transcription termination

Transcription termination is a tightly regulated, R-loop-mediated process that ensures release of the nascent transcript and polymerase recycling (Rodriguez-Molina et al, 2023; Xie et al, 2023). Recent studies have identified new factors involved in this process. The RNA helicase DDX21, together with METTL3, promotes m6A deposition on nascent transcripts at R-loop-prone regions, particularly at termination sites, thereby facilitating recruitment of the termination factor XRN2 (Hao et al, 2024).

A novel role in termination has also been uncovered for the elongation factor and histone chaperone SPT6. Independent of its canonical partner IWS1, SPT6 promotes transcription termination, likely by recruiting the polyadenylation factor PCF11 and the phosphatase PNUTS to RNAPII, as depletion of these factors leads to transcriptional readthrough (Bejjani et al, 2025).

By contrast, the role of Senataxin (SETX) in transcription termination has recently been re-evaluated. Acute SETX depletion revealed no major effects on termination of protein-coding genes, ncRNAs, PROMPTs or enhancer RNAs, challenging earlier models in human and yeast systems (Han et al, 2025; Porrua and Libri, 2013; Skourti-Stathaki et al, 2011; Xie et al, 2022). Instead, TT-seq and PRO-seq analyses suggest that SETX promotes early elongation by limiting RNAPII pausing and backtracking, with R-loop accumulation restricted to promoter-proximal regions rather than occurring globally (Crossley et al, 2023; Hatchi et al, 2015). Together, these findings highlight the need to reassess transcription termination mechanisms to fully understand how their regulation prevents TRCs at this critical step.

## TRCs cause local and global chromatin alterations

**Figure 4:**
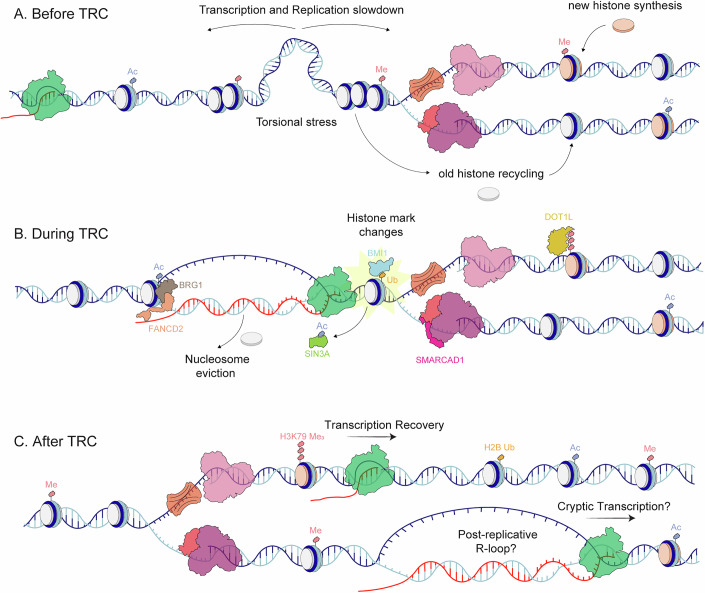
Chromatin dynamics during TRCs. (**A**) Topological stress, histone recycling and de novo histone synthesis are processes that generally slow both replication and transcription, thus they need to be tightly regulated to avoid collisions. (**B**) At sites of TRCs, multiple chromatin factors contribute directly or indirectly to conflict resolution by evicting nucleosomes, catalyzing histone modifications, remodeling surrounding chromatin, or facilitating R-loop/TRC resolution. (**C**) After TRCs are resolved, transcription recovers through re-establishment of epigenetic marks deposited by chromatin modifiers such as DOT1L. However, cryptic transcription and the accumulation of post-replicative R-loops may occur, requiring specialized regulatory pathways to re-establish the chromatin landscape and maintain transcriptional fidelity. See review text for more information on the roles of the depicted factors.

Both transcription and replication involve transient perturbation and restoration of chromatin structure. When replication fork progression and transcription are simultaneously obstructed by TRCs, this can induce chromatin alterations at specific genomic loci and drive epigenomic instability (Fig. 4A).

Recent studies highlight the interplay between chromatin and TRCs and suggest that local chromatin modifications are critical for stabilizing stalled replication forks and managing R-loop homeostasis, especially at TRC sites. For example, the methyl-CpG-binding domain protein MBD1 and the chromatin factor PSIP1/LEDGF recruit PARP1 to conflict sites. Loss of MBD1 leads to global R-loop accumulation, uncontrolled fork acceleration and DNA2-mediated degradation of nascent DNA (Yu et al, 2024), whereas PSIP1 depletion induces R-loop accumulation, transcriptional arrest and DNA damage at gene promoters (Jayakumar et al, 2024). Similarly, the SIN3A histone deacetylase complex prevents genomic instability by maintaining low levels of histone H3 acetylation at stalled forks (Fig. 4B). Without SIN3A, hyperacetylation leads to fork breakage and MRE11-dependent degradation (Munoz et al, 2024). Stalling of RNAPII itself can trigger local chromatin acetylation via p300, which relaxes chromatin and promotes R-loop formation. This signaling cascade activates ATM, which then further modulates chromatin by triggering p38MAPK/MSK1-dependent phosphorylation of H3S10 and H2AX (Salas-Armenteros et al, 2025).

The chromatin network further adapts through dynamic switching of modifications. Upon replication stress, decrotonylation of H2AK119 by SIRT1 is a prerequisite for subsequent ubiquitylation by BMI1. Accumulation of ubiquitylated H2AK119 at reversed forks triggers transcriptional repression and release of RNAPII, effectively attenuating TRCs (Fig. 4B) (Hao et al, 2022). Furthermore, R-loop-prone TRC sites were recently shown to drive nucleosome eviction and a specific increase in H3K79 methylation at R-loop sites. This epigenetic mark, deposited by the methyltransferase DOT1L, is essential for resolving conflicts and restoring transcriptional output to prevent global replication stress and DNA damage (Fig. 4B) (Werner et al, 2025). Conversely, increased turnover of the histone variant H2A.Z through upregulation of the H2A.Z-specific chaperone ANP32E in triple-negative breast cancers alters RNAPII processivity, leading to enhanced TRC formation and activation of an ATR-dependent DNA damage response, exposing a potential therapeutic vulnerability in this aggressive breast cancer type (Lago et al, 2025).

Besides histone modifications and variants, several other chromatin proteins have been implicated in TRC resolution. A recent correlative study of genome-wide datasets defined a network of chromatin factors enriched at potential R-loop-prone TRC sites, including specialized remodelers such as SWI/SNF, INO80 and BRG1 that may work independently or in concert to prevent TRCs from evolving into permanent mutagenic hotspots (Fig. 4B) (Bayona-Feliu et al, 2023). In addition, the chromatin remodeler SMARCAD1 has recently been proposed to have a dual function as a sensor of TRCs and R-loops, through its replisome-binding N-terminal domain, and as a resolver of conflicts via its catalytic C-terminal domain (Fig. 4B) (Uruci et al, 2024).

Beyond conflict resolution, RNAPII plays a global role in organizing chromatin post-replication. Although most histone modifications are uncoupled from transcription, RNAPII is required for the re-establishment of transcription-associated marks such as H3K36me2 and H4K16ac on nascent DNA (Fig. 4C). More importantly, RNAPII stabilizes the association of hundreds of chromatin remodeling enzymes and DNA repair proteins with newly replicated chromatin. Without active transcription following passage of the replication fork, recruitment of these factors is impaired, leaving nascent DNA vulnerable (Bandau et al, 2024).

Collectively, these findings illustrate that the chromatin landscape is both a target and a critical regulator of TRCs, with a network of histone modifiers, chaperones and remodelers dynamically orchestrating local and global epigenetic states to preserve fork stability and prevent genomic instability.

## Targeting TRCs as a therapeutic vulnerability in cancers

Recent advances have reframed TRCs from unavoidable by-products of genome metabolism into actionable therapeutic vulnerabilities across many cancers. Notably, the synthetic lethality of PARP inhibitors (PARPi) in HR-deficient tumors has been linked to defective repair of TRC-induced DNA damage. Contrary to the classical PARP-trapping model, PARP1, together with TIMELESS and TIPIN, protects the replisome from TRCs during early S phase (Petropoulos et al, 2024). Consistently, TRC-driven R-loop accumulation sensitizes spliceosome-mutant leukemias to PARP1 inhibition (Liu et al, 2024), and exacerbating these conflicts may overcome PARPi resistance. For example, combining PARPi with the HIF-1α inhibitor acriflavine hydrochloride increases R-loop-mediated collisions and DSBs in ovarian cancer cells (Lin et al, 2025). In line with this, a synthetic lethal siRNA screen in BRCA1-mutant cells identified transcription regulators, including MEPCE, LARP7 and the PAF1 complex, as essential for suppressing R-loop accumulation, TRCs and genome instability (Patel et al, 2023). Together, these findings establish excessive TRCs as a vulnerability in BRCA1-deficient cancers and highlight RNAPII elongation and R-loop homeostasis as therapeutic targets.

Pan-cancer analyses of 6193 whole-genome-sequenced tumors further implicate TRCs as drivers of distinct structural variation signatures, particularly large tandem duplications linked to CDK12 loss (Yang et al, 2024c). In metastatic prostate and upper gastrointestinal cancers, CDK12 inactivation, frequently co-occurring with TP53 mutations, promotes R-loop accumulation and harmful transcription-replication collisions (Tien et al, 2024).

Beyond CDK12, RNAPII regulation by CDK9 is also critical. The selective CDK9 inhibitor AZD4573 induces S phase TRCs and catastrophic DNA damage, especially in breast cancer models, with conflict resolution depending on the DEAD-box helicase DDX25 (Lee et al, 2025). These insights reveal clinically relevant vulnerabilities, as CDK12-deficient tumors show sensitivity to ATR, CHK1 or WEE1 inhibition and exhibit synthetic lethality with CDK13 loss. Accordingly, TRC-associated signatures may serve as prognostic biomarkers and guide precision oncology strategies.

Several therapeutic approaches now directly target TRCs. The PCNA inhibitor AOH1996 enhances interaction between PCNA and the RNAPII subunit RPB1, trapping transcription complexes on chromatin and inducing transcription-dependent DSBs (Gu et al, 2023). In multiple myeloma, high immunoglobulin transcription creates pronounced transcriptional stress, rendering malignant plasma cells particularly sensitive to the G-quadruplex stabilizer pyridostatin (Dutrieux et al, 2025). Similarly, extrachromosomal DNA drives extreme oncogene transcription, generating widespread TRCs and chronic replication stress that can be selectively exploited for cancer treatment with the CHK1 inhibitor BBI-2779 (Tang et al, 2024). Collectively, these studies position modulation of TRC formation and resolution - via checkpoint inhibition, G-quadruplex stabilization or PCNA targeting - as a promising avenue for cancer-selective therapies.

## Current methods and limitations to detect TRCs

A major limitation in studying TRCs is the lack of specialized tools to directly and unambiguously observe them (Zhu et al, 2025). Proximity ligation assays (PLA) using antibodies against elongating RNAPII-pS2 and PCNA are commonly used to quantify TRCs (<40 nm proximity) across cell types or conditions (Ke et al, 2025; Lalonde et al, 2024). However, because PLA relies on molecular proximity, it cannot assess conflict severity, detect transient encounters or capture TRCs with greater spatial separation between the transcription and replication machineries.

R-loop mapping has also been widely used as an indirect proxy to identify TRC sites, based on the assumption that their accumulation reflects underlying TRCs. However, this inference is complicated by the fact that R-loops can arise both as causes and consequences of replication stress, or in contexts unrelated to TRCs, and that a subset of detected signals may form ex vivo during chromatin deproteinization procedures, thereby potentially inflating R-loop quantification (Belotserkovskii and Hanawalt, 2022), reviewed in (Chedin et al, 2021; Crossley et al, 2019). In addition, commonly used tools such as the antibody S9.6, when applied in imaging-based techniques, require carefully controlled conditions to avoid confounding cross-reactivity. Newer engineered tools, including catalytically inactive RNaseH1-based probes and synthetic affinity reagents, offer robust alternatives, as reviewed in (Chedin et al, 2021; Crossley et al, 2019).

Recent advancements have shifted from indirect readouts towards real-time visualization and single-molecule analysis. The development of genetically encoded “mintbodies” allows tracking of RNAPII-pS2 in living cells, revealing that elongating complexes maintain spatial mobility distinct from DNA replication domains (Uchino et al, 2022). Live-cell imaging has further enabled real-time monitoring of replisome progression, uncovering a wave of transcriptional repression that precedes the fork to mitigate collisions (Tsirkas et al, 2022).

Direct visualization, however, remains technically demanding. Electron microscopy (EM) has successfully identified post-replicative RNA:DNA hybrids at HO-TRC sites (Fig. 4C) (Stoy et al, 2023), and atomic force microscopy (AFM) has detected direct interactions of proteins such as PARP1 and SMARCAD1 with R-loops (Laspata et al, 2023; Uruci et al, 2024). These methods are often labor-intensive and low-throughput. Single-molecule DNA combing assays can enable simultaneous analysis of replication fork progression and R-loop presence at individual DNA molecules. However, their spatial resolution does not distinguish R-loops located ahead of the replication fork from post-replicative RNA:DNA hybrids behind it, limiting mechanistic inference on whether these structures directly impede fork progression (Ivanov et al, 2024). Despite these innovations, a major limitation remains the difficulty of distinguishing TRC-associated structures from normal intermediates, such as Okazaki fragments.

While imaging and single-molecule techniques reveal the dynamics and frequency of TRCs, they cannot map their precise locations genome-wide. To address this, high-resolution sequencing approaches have been developed to identify endogenous TRC sites that remain invisible to imaging. TRIPn-seq identifies TRCs by sequentially immunoprecipitating RNAPII phosphorylated on Ser5 (pS5) together with nascent DNA, revealing that conflicts are highly enriched at TSSs and early-replicating regions characterized by G-quadruplexes and R-loops (St Germain et al, 2022). Notably, the use of an RNAPII pS5-specific antibody in this approach likely contributes to the observed enrichment at TSS, where promoter-proximal pausing and early elongation are predominant. Complementary to this, modified CUT&Tag profiling of topoisomerases has identified TOP3A as a potential sensor for both HO and CD-TRCs, particularly within the 5′ regions of genes (Zhang et al, 2024). Furthermore, engineering of enDR3 - a tandem fusion protein based on RNase H3 - offers a high-affinity alternative to the S9.6 antibody for capturing RNA:DNA hybrids that often form at TRC sites either as a cause or as a consequence (Jedynak-Slyvka et al, 2025).

Future single-molecule imaging approaches could further advance TRC detection. The ChromStretch assay, previously used to visualize chromatin composition and histone modifications at individual replication forks (Cherdyntseva et al, 2025; Duzanic et al, 2025; Gaggioli et al, 2023; Galloy et al, 2025), could be adapted to co-detect RNAPII at forks alongside histone marks, enabling analysis of chromatin dynamics during TRCs. Complementary proteomics strategies could uncover novel pathways and interactors. Proximity biotin-labeling offers a promising strategy to detect TRCs. For example, the UltraID biotin ligase (Kubitz et al, 2022; van Schie et al, 2026), could be fused to transcription elongation factors or RNAPII subunits to selectively detect HO or CD-TRCs. An intriguing alternative is split-TurboID (Cho et al, 2020), which becomes active only when its two fragments reconstitute upon close proximity. This approach would enable pairing RNAPII subunits with PCNA or CMG subunits, thereby labeling proteins involved in conflict resolution specifically when the transcription and replication machineries approach one another.

## Concluding remarks and future perspectives

TRCs have emerged as central determinants of genome integrity through their influence on RNAPII and nascent RNA, DNA repair and chromatin architecture. Despite major advances in recent years, our understanding of TRC dynamics remains incomplete (Box 1). A key outstanding question is what precisely constitutes a TRC. How can we distinguish a true conflict between the replisome and RNAPII from the physiological proximity of these processes? Currently, there is no consensus on how close the two machineries must be to define a TRC event versus non-harmful transcription and replication occurring in parallel.

Moreover, promoter-proximal sites, RNAPII backtracking and transcription termination have all been shown to modulate TRCs, but how the cell prioritizes which polymerase to remove and at which stage of the transcription cycle remains unclear. This gap is particularly notable given the replication-centric approaches that many previous studies have relied upon, leaving the interplay with replication forks in vivo largely unexplored.

The notion that only HO-TRCs are deleterious has been challenged by the discovery of CD-specific pathways and the evidence of evolutionary selection for CD-TRCs. Further studies are needed to uncover pathways selective for different TRC types and to explore how HO and CD-TRC resolution pathways interact. In addition, the strict classification of HO versus CD conflicts based solely on gene orientation may be oversimplified; HO-like effects could arise when a backtracked, co-directional RNAPII encounters an incoming replisome, offering a potential explanation for contrasting observations regarding CD-TRC severity.

The chromatin environment appears to act both as a target and a regulator of TRCs. Many questions remain regarding the dynamics and spatiotemporal coordination among chromatin remodeling, transcription pausing and elongation, R-loop accumulation and fork progression. Investigating three-dimensional chromatin reorganization during TRCs and the resulting epigenetic plasticity could reveal cell-type-specific vulnerabilities to these conflicts.

Technological limitations remain a significant bottleneck. Developing methods for genome-wide mapping of TRCs at single-molecule resolution, for example, using emerging long-read sequencing technologies, would greatly enhance our understanding of TRC occurrence, their mutational consequences and their contributions to human disease, particularly cancer. As clinical strategies increasingly aim to target TRCs as a druggable therapeutic axis, a deeper mechanistic understanding of TRCs, including the interplay and potential redundancy of various resolution pathways, will be essential to more accurately predict which tumors are likely to respond to specific interventions.

## Supplementary information

Peer Review File
