# Supplementary material for: Polymerase face-off: emerging concepts in transcription-replication coordination
Source: EMBO Rep. 2026 May 15;27(12):3157–72. doi: 10.1038/s44319-026-00800-w (PMC13303910; doi:10.1038/s44319-026-00800-w)
Supplement: Supplementary file 1 — Peer Review File [file 44319_2026_800_MOESM1_ESM.pdf]

EMBO reports

Polymerase face-off: emerging concepts in transcription-replication coordination

Sidrit Uruci, Maxime Lalonde, Martijn Luijsterburg, and Stephan Hamperl

Corresponding author(s): Stephan Hamperl (stephan.hamperl@helmholtz-muenchen.de)

Review Timeline:	Submission Date:	11th Mar 26
	Editorial Decision:	15th Apr 26
	Revision Received:	19th Apr 26
	Accepted:	7th May 26

Editor: Esther Schnapp

Transaction Report:

(Note: With the exception of the correction of typographical or spelling errors that could be a source of ambiguity, letters and reports are not edited. Depending on transfer agreements, referee reports obtained elsewhere may or may not be included in this compilation. Referee reports are anonymous unless the Referee chooses to sign their reports.)

Dear Stephan,

Thank you for the submission of your Review to EMBO reports. We have now received the comments from 2 referees while a third referee has not yet accepted to review your ms. Given that referee 2's report is very good, I am making a decision now based on the 2 reports we have and in the interest of time.

As you will see, both referees acknowledge that the review is interesting, timely, well-written, distinct and valuable, which is great. Referee 2 also has several suggestions for how the review could be further improved, and I think all suggestions are good and should be addressed, if you agree, of course.

I like the figures and would suggest that we use them as they are, but if you prefer, we can also ask our graphics designer to redraw the figures. This will take approx 10 working days. If you used BioRender or similar to prepare the figures (and they will not be redrawn), this needs to be mentioned in the figure legends.

As for timing, would it be possible for you to submit the revised version of the review in the first week of May? If you need more time please just let me know.

I think this is a very nice review and I am looking forward to seeing this published!

With best wishes,
Esther

Esther Schnapp, PhD
Senior Editor
EMBO reports

Referee #1:

In this review the authors have addressed the complexity of the coordination between transcription and replication. I like in particular the fact that the this complexity has been analyzed in a holistic way, not only looking at transcription at an impairment to DNA replication leading to increased genome instability, but also the fact that both transcription and replication occur on chromatin, hence positive and negative crosstalks between these processes need to be assessed from all sides. Indeed, this review presents an up-to-date picture of what we know in the field, but also of what the current limitations to our understanding are, both in terms of limitations with current approaches but also conceptually. I particularly like in this sense when in the concluding remarks ask to what is precisely a TRC, as proximity or overlap does not necessarily mean "bad" or DNA damage. All in all, I am in favour of this review being published as it is. The references are complete, the problem is dissected in easy to read paragraphs that focus on specific aspects of the problem, and the figures, although simple in their design, make specific aspects described in the text easy to understand.

Referee #2:

This timely and well-written review article addresses transcription-replication conflicts (TRCs) as a major source of genetic and epigenetic instability in eukaryotic cells. Although several recent reviews have covered this area, the present manuscript offers a distinctive and valuable perspective by focusing on the direct impact of RNA polymerases on replication fork progression, rather than primarily emphasizing R-loop-mediated mechanisms. The authors present a comprehensive and up-to-date synthesis of the literature, including very recent studies, and effectively convey the mechanistic complexity and diversity of TRCs across systems. The manuscript is nuanced and avoids oversimplification, which will be appreciated by specialists while remaining accessible to a broader audience. In particular, the sections on backtracking dependent conflicts (BD TRC) and on the impact of codirectional collisions are particularly interesting. The manuscript also highlights emerging links between TRCs and human diseases, particularly cancer, and discusses potential therapeutic applications. The figures are clear, well-designed, and effectively support the text. Overall, this review should be of interest to a broad readership spanning DNA replication, transcription, DNA repair and chromatin biology. However, several points should be addressed to further strengthen the manuscript.

Introduction: The statement "The diploid human genome comprises over 6 billion base pairs and approximately 20,000 protein-coding genes" is somewhat misleading in this context. If the diploid genome is considered, the number of potentially interfering genes should also be doubled. Moreover, the potential contribution of pervasive transcription to TRCs should be discussed.

Section 2.1: In the Introduction, the authors refer to "their timely displacement from the DNA template" for both replication and transcription machineries. However, this type of displacement is effectively restricted to RNA polymerases, since the replisome

is not subject to the same regulatory eviction mechanisms. The authors should clarify this point and explicitly emphasize that RNAPII (and not the replication machinery) is the entity being displaced. Furthermore, the review should briefly summarize the mechanisms that promote RNAPII displacement at head-on transcription-replication conflicts, including the roles of checkpoint kinases (Mec1/ATR), the INO80 chromatin-remodeling complex, and the segregase p97. These pathways operate not only at promoters but also across gene bodies, where head-on collisions are most likely to stall replication forks and generate genomic instability. Mentioning these players in the main text of Section 2.1, rather than confining them to promoter-associated regulation, would better reflect their broader impact on TRC resolution.

Section 2.1: Transcription-Replication Coordination. Two important concept are currently missing. First, transcription is inherently discontinuous, occurring in bursts separated by inactive periods (PMID: 30554876). This may provide temporal windows during which replication forks can traverse highly-expressed genes with reduced risk of conflict. Second, replication origins are preferentially positioned upstream of active genes, which helps minimize head-on collisions. At least one of the following studies should be cited: PMID: 26751768, 30598550, 32769985.

Section 2.1: The observation that "actively transcribing RNA polymerases can push multiple MCM helicases from licensed origins over long distances" is particularly relevant for preventing initiation within gene bodies. This displacement of MCMs by RNA polymerases was first demonstrated in vivo in yeast (PMID: 26656162). Of note, the Halazonetis lab showed that oncogene-induced shortening of G1 promotes the firing of intergenic origins in cancer cells, which may relate to this process (PMID: 29466339).

Section 2.2: Although R-loops are not the primary focus of this review, they remain central to the field. It has been suggested that a subset of R-loops detected by standard mapping approaches may form ex-vivo following chromatin deproteinization (PMID: 35074657). It would therefore be valuable to briefly discuss technical limitations in R-loop mapping and highlight best practices, as outlined in PMID: 33411340.

Section 2.3: The statement "RNAPII has been shown to also transiently form RNA:DNA hybrids at stalled forks to safeguard nascent DNA from uncontrolled degradation by the DNA2 nuclease ... (Song et al, 2025)" should be tempered. This article does not provide direct evidence that RNAPII acts at stalled forks to generate hybrids. Notably, a companion study in the same issue of Mol Cell (PMID: 39706185) reports similar observations but proposes a different mechanistic interpretation (PMID: 39706185). In addition, post-replicative RNA:DNA hybrids have been shown to impair fork restart in the absence of RNaseH2 (PMID: 37855233).

Section 3.2: RECQL5 is repeatedly misspelled in this paragraph and in Fig. 3C.

Section 6: The statement "Single-molecule DNA combing assays now allow simultaneous detection of R-loops and replication forks, providing direct evidence that these hybrids inhibit fork progression (Ivanov et al, 2024)" appears too strong. The resolution of this does not allow discrimination between R-loops located ahead of the fork and post-replicative RNA:DNA hybrids behind it, making it difficult to conclude that these structures directly impede fork progression.

Section 6: Regarding the sentence "TRIPn-seq identifies TRCs by sequentially immunoprecipitating phosphorylated RNAPII and nascent DNA, revealing that conflicts are highly enriched at TSSs and early-replicating regions characterized by G-quadruplexes and R-loops (St Germain et al, 2022)." it should be specified that this study uses an antibody against RNAPII phosphorylated on Ser5 (pS5), which likely explains why conflicts are predominantly detected at transcription start sites.

Referee #1:

In this review the authors have addressed the complexity of the coordination between transcription and replication. I like in particular the fact that the this complexity has been analyzed in a holistic way, not only looking at transcription at an impairment to DNA replication leading to increased genome instability, but also the fact that both transcription and replication occur on chromatin, hence positive and negative crosstalks between these processes need to be assessed from all sides. Indeed, this review presents an up-to-date picture of what we know in the field, but also of what the current limitations to our understanding are, both in terms of limitations with current approaches but also conceptually. I particularly like in this sense when in the concluding remarks ask to what is precisely a TRC, as proximity or overlap does not necessarily mean "bad" or DNA damage.
All in all, I am in favour of this review being published as it is. The references are complete, the problem is dissected in easy to read paragraphs that focus on specific aspects of the problem, and the figures, although simple in their design, make specific aspects described in the text easy to understand.

We are very grateful to the reviewer for their kind words and recommendation for publication. We are pleased that our discussion on the complexity of chromatin-mediated crosstalk and our conceptual questioning of what defines a TRC were well-received. We also thank the reviewer for the positive feedback regarding the figures and the readability of the text.

Referee #2:

This timely and well-written review article addresses transcription-replication conflicts (TRCs) as a major source of genetic and epigenetic instability in eukaryotic cells. Although several recent reviews have covered this area, the present manuscript offers a distinctive and valuable perspective by focusing on the direct impact of RNA polymerases on replication fork progression, rather than primarily emphasizing R-loop-mediated mechanisms. The authors present a comprehensive and up-to-date synthesis of the literature, including very recent studies, and effectively convey the mechanistic complexity and diversity of TRCs across systems. The manuscript is nuanced and avoids oversimplification, which will be appreciated by specialists while remaining accessible to a broader audience. In particular, the sections on backtracking dependent conflicts (BD TRC) and on the impact of codirectional collisions are particularly interesting. The manuscript also highlights emerging links between TRCs and human diseases, particularly cancer, and discusses potential therapeutic applications. The figures are clear, well-designed, and effectively support the text. Overall, this review should be of interest to a broad readership spanning DNA replication, transcription, DNA repair and chromatin biology. However, several points should be addressed to further strengthen the manuscript.

We really appreciate the positive feedback from the reviewer and are thankful for insightful suggestions to strengthen the manuscript.

1. **Introduction:** The statement "The diploid human genome comprises over 6 billion base pairs and approximately 20,000 protein-coding genes" is somewhat misleading in this context. If the diploid genome is considered, the number of potentially interfering genes should also be doubled.

   We thank the reviewer for pointing this out and agree that in the context of TRCs, the biallelic nature of the genome is critical as it doubles the number of potential interference sites. We have revised the text to specify that the diploid genome contains approximately 40,000 protein-coding gene copies (alleles). The sentence now reads as follows:

“The diploid human genome comprises over 6 billion base pairs and approximately 40,000 protein-coding gene copies, representing two alleles of the ~22,000 unique genes (Venter et al, 2001)”

2. Moreover, the potential contribution of pervasive transcription to TRCs should be discussed.

   We thank the reviewer for raising this point. Indeed, TRCs can happen outside of canonical protein-coding genes, raising substantially the amount of potential transcription units that can interfere with replication. To address this point, we added this sentence in the introduction:

   “Moreover, as a result of pervasive intergenic and non-coding transcription, nearly 85% of the genome exhibits detectable transcriptional activity, albeit at levels generally lower than those of protein-coding genes (Hangauer et al, 2013). “

3. **Section 2.1**: In the Introduction, the authors refer to "their timely displacement from the DNA template" for both replication and transcription machineries. However, this type of displacement is effectively restricted to RNA polymerases, since the replisome is not subject to the same regulatory eviction mechanisms. The authors should clarify this point and explicitly emphasize that RNAPII (and not the replication machinery) is the entity being displaced.

   We thank the reviewer for highlighting this important distinction. We agree that regulatory eviction and timely displacement are mechanisms specifically targeted at RNA polymerases to prevent interference, whereas the replisome is not subject to the same displacement kinetics. We have revised Section 2.1 to explicitly clarify that RNAPII is the entity being displaced to safeguard the progression of the replication fork. Please refer to our response under point 5 to see the relevant modification made in that section, encompassing points 3,4, and 5.

4. Furthermore, the review should briefly summarize the mechanisms that promote RNAPII displacement at head-on transcription-replication conflicts, including the roles of checkpoint kinases (Mec1/ATR), the INO80 chromatin-remodeling complex, and the segregase p97. These pathways operate not only at promoters but also across gene bodies, where head-on collisions are most likely to stall replication forks and generate genomic instability. Mentioning these players in the main text of **Section 2.1**, rather than confining them to promoter-associated regulation, would better reflect their broader impact on TRC resolution.

   We thank the reviewer for this suggestion, and we now discuss the involvement of Mec1, INO80 and PAF1 complex in removing RNAPII from collisions sites (Poli *et al*, 2016). See point 5 to see the relevant modification made in that section, encompassing points 3,4, and 5.

5. **Section 2.1:** Transcription-Replication Coordination. Two important concept are currently missing. First, transcription is inherently discontinuous, occurring in bursts separated by inactive periods (PMID: 30554876). This may provide temporal windows during which replication forks can traverse highly-expressed genes with reduced risk of conflict. Second, replication origins are preferentially positioned upstream of active genes, which helps minimize head-on collisions. At least one of the following studies should be cited: PMID: 26751768, 30598550, 32769985.

   We apologize for omitting these studies that reveal important core features that help coordinate transcription and replication. They are now mentioned accordingly in section 2.1 as such (together with the modification made based on point 3 and 4):

"The widespread nature of transcription and replication requires extensive spatial and temporal coordination of the two machineries to prevent TRCs and maintain genome integrity (Fig. 2A). The inherent bursting dynamics of transcription (Rodriguez *et al*, 2019) help mitigate the risk of TRCs by introducing intermittent periods of reduced transcriptional activity. Similarly, the preferential positioning of replication origins upstream of active genes further limits head-on collisions (Chen *et al.*, 2019; Petryk *et al*, 2016; Promonet *et al*, 2020). Despite these spatial and temporal safeguards, such mechanisms are insufficient to fully prevent conflicts. Indeed, recent studies highlight multi-layered "traffic management" at different genomic scales, involving spatial confinement into distinct compartments, modulation of elongation kinetics of the two machineries and, where necessary, the timely stabilization and remodelling of replication forks and the displacement of RNAPII from the DNA template. At collision sites, this process is promoted by checkpoint signalling and chromatin remodelling pathways, including Mec1/ATR-dependent checkpoint activation, the INO80 chromatin remodelling complex, and the p97 segregase, which collectively facilitate RNAPII removal (Poli *et al*, 2016)."

6. **Section 2.1:** The observation that "actively transcribing RNA polymerases can push multiple MCM helicases from licensed origins over long distances" is particularly relevant for preventing initiation within gene bodies. This displacement of MCMs by RNA polymerases was first demonstrated in vivo in yeast (PMID: 26656162). Of note, the Halazonetis lab showed that oncogene-induced shortening of G1 promotes the firing of intergenic origins in cancer cells, which may relate to this process (PMID: 29466339).

   We thank the reviewer for acknowledging the importance of this mechanism. We now cite the recommended references to further emphasize this point. The section now reads as follows:

   ´To mitigate these risks, the genome exhibits remarkable plasticity. Actively transcribing RNA polymerases can push multiple MCM helicases from licensed origins over long distances, even in the context of chromatin (Scherr *et al*, 2022). This phenomenon, first demonstrated *in vivo* in yeast (Gros *et al*, 2015) , likely contributes to the deregulated firing of intergenic origins observed in cancer cells upon oncogene-induced shortening of G1-phase (Macheret & Halazonetis, 2018) . This mechanism provides a simple but powerful way to redistribute origins of replication away from highly transcribed genes, thereby limiting initiation within gene bodies."

7. **Section 2.2**: Although R-loops are not the primary focus of this review, they remain central to the field. It has been suggested that a subset of R-loops detected by standard mapping approaches may form ex-vivo following chromatin deproteinization (PMID: 35074657). It would therefore be valuable to briefly discuss technical limitations in R-loop mapping and highlight best practices, as outlined in PMID: 33411340.

   We thank the reviewer for this suggestion. While we have intentionally kept the focus on the broader aspects of transcription-replication coordination, we agree that R-loops are frequently used as a proxy for TRCs and that their detection comes with significant technical caveats. Accordingly, we have added a dedicated subsection in Section 6 (Current Methods and Limitations) to discuss R-loop mapping limitations. This includes the potential for ex vivo R-loop formation following deproteinization and a discussion of the 'best practices' required to ensure data reliability, citing the seminal work suggested by the reviewer.

   "R-loop mapping has also been widely used as an indirect proxy to identify TRC sites, based on the assumption that their accumulation reflects underlying TRCs. However, this inference is complicated by the fact that R-loops can arise both as causes and consequences of replication

stress, or in contexts unrelated to TRCs, and that a subset of detected signals may form ex vivo during chromatin deproteinization procedures, thereby potentially inflating R-loop quantification (Belotserkovskii & Hanawalt, 2022), reviewed in (Chedin et al, 2021; Crossley et al, 2019). In addition, commonly used tools such as the antibody S9.6, when applied in imaging-based techniques, require carefully controlled conditions to avoid confounding cross-reactivity. Newer engineered tools, including catalytically inactive RNaseH1-based probes and synthetic affinity reagents offer robust alternatives, as reviewed in (Chedin et al., 2021; Crossley et al., 2019)."

8. **Section 2.3**: The statement "RNAPII has been shown to also transiently form RNA:DNA hybrids at stalled forks to safeguard nascent DNA from uncontrolled degradation by the DNA2 nuclease ... (Song et al, 2025)" should be tempered. This article does not provide direct evidence that RNAPII acts at stalled forks to generate hybrids. Notably, a companion study in the same issue of Mol Cell (PMID: 39706185) reports similar observations but proposes a different mechanistic interpretation (PMID: 39706185). In addition, post-replicative RNA:DNA hybrids have been shown to impair fork restart in the absence of RNaseH2 (PMID: 37855233).

   We thank the reviewer for bringing this to our attention. We tempered the conclusions from Song et al 2025 and cited the companion study. The section now read as such:

   "Interestingly, while R-loops are often genotoxic, R-loop formation at stalled forks has been suggested to contribute to protection of nascent DNA from DNA2-mediated degradation (Song *et al*, 2025). However, in an alternative model the R-loops at stalled forks facilitate DNA2-mediated DNA resection and allow proper fork restart (Xu *et al*, 2025). In line with this model, post-replicative R-loops have also been implicated in impairing fork restart in RNase H2-deficient contexts (Heuze *et al*, 2023)."

9. **Section 3.2**: RECQL5 is repeatedly misspelled in this paragraph and in Fig. 3C.

   We have modified the text accordingly.

10. **Section 6**: The statement "Single-molecule DNA combing assays now allow simultaneous detection of R-loops and replication forks, providing direct evidence that these hybrids inhibit fork progression (Ivanov et al, 2024)" appears too strong. The resolution of this does not allow discrimination between R-loops located ahead of the fork and post-replicative RNA:DNA hybrids behind it, making it difficult to conclude that these structures directly impede fork progression.

    The reviewer is correct and we have modified the text to improve accuracy and tone down the statement. It now reads as such:

    "Single-molecule DNA combing assays can enable simultaneous analysis of replication fork progression and R-loop presence at individual DNA molecules. However, their spatial resolution does not distinguish R-loops located ahead of the replication fork from post-replicative RNA:DNA hybrids behind it, limiting mechanistic inference on whether these structures directly impede fork progression (Ivanov *et al*, 2024)."

11. **Section 6:** Regarding the sentence "TRIPn-seq identifies TRCs by sequentially immunoprecipitating phosphorylated RNAPII and nascent DNA, revealing that conflicts are highly enriched at TSSs and early-replicating regions characterized by G-quadruplexes and R-loops (St Germain et al, 2022)." it should be specified that this study uses an antibody against

RNAPII phosphorylated on Ser5 (pS5), which likely explains why conflicts are predominantly detected at transcription start sites.

We thank the reviewer for pointing this out which we have now included in the manuscript as such:

"TRIPn-seq identifies TRCs by sequentially immunoprecipitating RNAPII phosphorylated on Ser5 (pS5) together with nascent DNA, revealing that conflicts are highly enriched at TSSs and early-replicating regions characterized by G-quadruplexes and R-loops (St Germain *et al*, 2022). Notably, the use of an RNAPII pS5-specific antibody in this approach likely contributes to the observed enrichment at transcription start sites, where promoter-proximal pausing and early elongation are predominant."

Dr. Stephan Hamperl
Helmholtz Zentrum München
Institute of Epigenetics and Stem Cells
Feodor-Lynen-Str. 21
München 81377
Germany

Dear Stephan,

I am pleased to inform you that your Review has been accepted for publication in EMBO reports. Your manuscript will be processed for publication by EMBO Press. It will be copy edited and you will receive page proofs prior to publication. Please note that you will be contacted by Springer Nature Author Services to complete licensing information.

No publication charges apply but please let us know should you be asked to pay.

If you have any questions, please do not hesitate to contact the Editorial Office. Thank you for your contribution to EMBO Reports.

Best regards,
Esther

Esther Schnapp, PhD
Senior Editor
EMBO reports

>>> Please note that it is EMBO Reports policy for the transcript of the editorial process (containing referee reports and your response letter) to be published as an online supplement to each paper. If you do NOT want this, you will need to inform the Editorial Office via email immediately. More information is available here: https://link.springer.com/partners/embo-press/editorial-policies#Peer%20review
